# Predictors for quitting smoking in smoking cessation clinics among female smokers in China

**DOI:** 10.18332/tid/159132

**Published:** 2023-02-17

**Authors:** Bingliang Lin, Li Xie, Xiaoyun Xie, Yongfu Yan, Luge Zhang, Lin Xiao

**Affiliations:** 1 Tobacco Control Office, Chinese Center for Disease Control and Prevention, Beijing, People’s Republic of China

**Keywords:** female smokers, smoking cessation clinic, quitting rate, predictors of quitting

## Abstract

**INTRODUCTION:**

The number of cessation clinics in China have been increasing ever since the Chinese government supported the establishment of smoking cessation clinics (SCCs) in each province in 2014. Many studies have examined smoking cessation behaviors among male smokers, but few of female smokers. This study aimed to understand female smokers’ quitting behaviors in SCCs and identify predictors of successful cessation.

**METHODS:**

This study used data of the SCCs Platform in China from 2018 to 2020. The self-reported 7-day point prevalence of abstinence rate (PPAR) at 1 month and at 3 months follow-up and the continuous abstinence rate (CAR) at 3 months follow-up are reported based on smokers’ characteristics and intention to treat analysis. A multiple logistic regression model was used to identify predictors of continuous abstinence at 3 months follow-up.

**RESULTS:**

The 7-day PPAR of female outpatients in SCCs was 29.20% at 1 month follow-up and 28.36% at 3 months follow-up. The CAR at 3 months follow-up was 19.88%. Female smokers who were prepared to quit within 7 days (AOR=2.86; 95% CI: 1.53–5.32), today (AOR=4.01; 95% CI: 2.35–6.85), had started to quit (AOR=7.11; 95% CI: 4.12–12.27), and used a combination of counseling and drugs (AOR=2.41; 95% CI: 1.73–3.35) were more likely to quit smoking. Associated with lower quitting rates were: living in the central region of China (AOR=0.47; 95% CI: 0.31–0.73) and the west region (AOR=0.48; 95% CI: 0.31–0.73); being aged 30–39 years (AOR=0.39; 95% CI: 0.23–0.64), and 40–49 years (AOR=0.41; 95% CI:0.24–0.69); being unemployed (AOR=0.64; 95% CI: 0.45–0.91); having a fair perceived health status at the first visit (AOR=0.65; 95% CI: 0.47–0.91) and a poor one (AOR=0.37; 95% CI: 0.21–0.64); having a moderate nicotine dependence (AOR=0.64; 95% CI: 0.44–0.92) and a severe one (AOR=0.50; 95% CI: 0.34–0.72).

**CONCLUSIONS:**

In our study, the region of residence, age, employment, perceived health status, Fagerström test for nicotine dependence (FTND), readiness to quit, and intervention model were independent predictors of quitting for female smokers. Improving the motivation to quit, providing intensive psychological interventions and equipping SCCs with cessation medication would assist female smokers to quit.

## INTRODUCTION

Tobacco use among women has become a public health issue^1^. There are 1.14 billion smokers worldwide and approximately 190 million were female smokers in 2019^2^. Previous studies indicate that females can develop nicotine dependence in a shorter time than males^3^. Among women, the top three global causes of death (cardiovascular disease, cancer, and chronic respiratory disease) are related to tobacco use^4^. In addition, female smokers have a higher risk of chronic obstructive pulmonary disease and coronary heart disease than male smokers at almost all smoking frequencies, and their unique physiological structure increases the risk of cervical cancer, breast cancer, and other diseases^5,6^. Furthermore, there is sufficient evidence that maternal smoking may lead to an ectopic pregnancy, miscarriage, and low birth weight of newborns during pregnancy^6^, which eventually causes decreased fertility rates.

The 2018 Global Adult Tobacco Survey (GATS) in China^7^ showed that among adult females, the smoking rate was 2.1%, average daily smoking was 11.3 cigarettes, and 8.5% intended to quit smoking within one month, and 23.5% attempted to quit smoking. Although the overall smoking rate is low, there are still 11.8 million adult female smokers, accounting for about 6.2% of the world’s female smokers due to China’s large population base. According to the results of China’s Internet tobacco marketing data in 2018^8^, teenagers and women have become the main targets of tobacco industry marketing. Consequently, female smoking warrants attention.

Smoking cessation is the quickest and most direct way to decrease the smoking rate^9^. Compared with other health interventions, quitting smoking is more health cost-effective^10^ and is beneficially achieved at any time^11^. A prospective study of 1.2 million women in Britain showed that female smokers who quit before 40 and 30 years of age could avoid more than 90% and more than 97% of their excess mortality, respectively^12^. Many studies showed that gender difference plays a critical role in smoking cessation^13^, with women having more difficulty quitting than men^14^. Evidence indicates that professional cessation services can increase quit rates^15^. The World Health Organization has identified tobacco dependence as a chronic addictive disease^16^, and only 3–5% of smokers can achieve long-term abstinence without professional help^17^. Since 2014, the Chinese government has started to support three smoking cessation clinics (SCCs) in each province per year to offer smoking cessation counselling and medication for smokers^18^.

In China, SCCs are located within hospitals in each province. For each SCC, one or two trained physicians provide cessation services to outpatients, including smoking cessation counselling and cessation drugs. These physicians usually come from respiratory medicine departments and have been certified by an expert panel in each province. The SCCs Platform was established by the Chinese Center for Disease Control and Prevention (China CDC) in 2018, which comprises 355 SCCs across the country. Despite several studies in China examining the determinants of successful smoking cessation among male smokers^19,20^, only some are about female smokers.

This study aims to understand the characteristics, tobacco usage and quitting behaviors of female smokers attending SCCs, and identify the factors influencing the result of quitting through the analysis of data from 355 SCCs in 2018–2020.

## METHODS

### Data source and study design

This study included 1534 female smokers who were offered smoking cessation services by medical staff at 355 SCCs nationwide, from 2018–2020. The data were collected by local physicians of each SCC using standardized and structured questionnaires designed by the tobacco control office of the China CDC at baseline, and at follow-up at 1 month and at 3 months. A cross-sectional study design was employed to describe female smokers’ quitting behaviors in SCCs and identify predictors of successful cessation. This in-depth analysis based on data from the SCCs platform was approved by the China CDC Institutional Review Board.

The questionnaire of the baseline survey consisted of questions on demographic characteristics, weight, height, blood pressure, smoking history, Fagerström test for nicotine dependence (FTND)^21^, quitting attempts, quit intention and reason, perceived confidence, difficulty and importance in quitting, perceived current health status, and smoking cessation drugs use. In the follow-up questionnaire, topics on withdrawal symptoms, relapsing and side effects of the drugs were added. In these SCCs, smoking cessation drugs included bupropion hydrochloride, varenicline, smoking cessation pills, and Chinese Medicine. The follow-up was conducted by face-to-face or telephone interview at 1 and at 3 months, from the day that an outpatient took action to quit. If there was no response after five interview calls on different days and times or if refused by an outpatient, the case was considered closed.

### Intervention

At the first visit, physicians assessed the tobacco use-related behaviors of female smokers using a baseline questionnaire through a 10-minute face-to-face interview. During this process, smokers can reflect on their smoking and quitting experiences. Then, all smokers were provided smoking cessation counselling for 30–40 minutes.

The counselling sessions were designed based on the Prochaska transtheoretical model^22^, which includes the 5As (ask, advise, assess, assist, arrange) and 5Rs (relevance, risks, rewards, roadblocks, repetition)^23^ methods. The 5As were applied to assess the stage of readiness in quitting smoking, and the 5Rs to strengthen smokers’ motivation to quit. In addition, physicians utilized the skills and methods to overcome psychological dependence and social-cultural factors related to smoking relapse. In general, physicians provided advice about the use of cessation drugs for all smokers, especially those with severe nicotine dependence or poor confidence. However, the outpatients decided whether to use the cessation drugs or not.

### Outcomes

This study used the self-reported continuous abstinence for 3 months as the primary outcome. Self-reported outcomes determined quit success without biochemical validation. The secondary outcomes were the self-reported 7-day point prevalence abstinence at follow-up at 1 month and at 3 months. The self-reported continuous abstinence rate (CAR) was defined as not smoking cigarettes within the past 3 months, and the self-reported 7-day point prevalence abstinence rate (PPAR) refers to no smoking behavior during the past seven days at 1 month and at 3 months following the decision to quit date. Smoking a whole or a puff of a cigarette was defined as a failure. Those who could not be followed up were regarded as smokers.

The level of nicotine addiction was based on the Fagerström test for nicotine dependence (FTND), and the level was classified as low (0–3), moderate (4–5), and severe (6–10)^21^. Body mass index (BMI, kg/m^2^) was calculated and categorized into underweight (<18.5), normal (18.5–24), overweight (24–28), and obese (≥28)^24^. The perceived confidence, difficulty, and importance of quitting were measured using a 0–10 Likert scale, with 0 indicating the lowest and 10 indicating the highest.

### Statistical analysis

The data were extracted from the SCCs platform to Excel software, and all analyses were performed through SAS version 9.4. Descriptive statistics were used to describe the baseline characteristics of female smokers, and the t-test was used to examine whether the data conformed to a normal distribution. We performed chi-squared test analyses to compare the effect of different baseline factors on quitting rate. The PPAR and CAR were calculated using intention to treat analysis (ITT). For the prevalence of continuous abstinence at follow-up at 3 months, we used multiple logistic regression analyses to explore the predictors of quitting under ITT and per-protocol (PP) methods and estimated adjusted odds ratios (AORs) and 95% confidence intervals (CIs). Odds ratios were adjusted for demographic characteristics and tobacco use-related factors. Demographic characteristics included region of residence, age, education level, occupation, and perceived health status. Tobacco use-related factors included the number of cigarettes smoked per day, FTND, the experience of quitting, readiness to quit, intervention method, perceived importance, difficulty, and confidence in quitting. Since the number of cigarettes smoked per day was a component in FTND, we only used FTND, a more comprehensive indicator, in the stepwise logistic regression model. All p values were two-tailed, and a p<0.05 indicated statistical significance.

## RESULTS

### Demographic characteristics

There were 1534 female smokers with a follow-up rate of 75.2% (1154/1534) at 1 month and 58.9% (903/1534) at 3 months. Table 1 shows that most female outpatients resided in the east region of China (61.2%). One-third were aged ≥ 60 years (31.6%), and 43.3% were college graduates or higher. Among the outpatients, 90.35% started smoking at ≥18 years of age, and 90.6% smoked <20 cigarettes per day. About 60.1% of outpatients smoked <21 years, and 61.1% never attempted quitting before the SCC visit. Less than half had a low nicotine dependence (38.53%), wanted to quit on the day when they came to the cessation clinics (28.2%), quit as a result of the concern about their health or that of their family members (40.2%), chose counselling combined with drugs (32.8%), and had a fair health condition (45.8%). In addition, the median scores of perceived importance, difficulty, and confidence in quitting were 8.0, 8.0, and 6.0, respectively (Table 1).

**Table 1:** Self-reported 7-day point prevalence abstinence rate (PPAR) of Chinese female smokers at follow-up at 1 month and at 3 months, according to demographic characteristics and tobacco-related factors (N=1534)

	*Smokers n (%)*	*PPAR at 1 month n (%)*	*χ^2^*	*p*	*PPAR at 3 months n (%)*	*χ^2^*	*p*
**Total**	1534 (100)	448 (29.20)			435 (28.36)		
**Demographic characteristics**							
**Region**			10.82	0.005		16.79	<0.001
East	939 (61.21)	301 (32.06)			294 (31.31)		
Central	278 (18.12)	75 (26.98)			52 (18.71)		
West	317 (20.67)	72 (22.71)			89 (28.08)		
**Age** (years)			6.55	0.161		6.13	0.190
18–29	178 (11.60)	56 (31.46)			58 (32.58)		
30–39	319 (20.80)	83 (26.02)			88 (27.59)		
40–49	255 (16.62)	63 (24.71)			60 (23.53)		
50–59	298 (19.43)	96 (32.21)			94 (31.54)		
≥60	484 (31.55)	150 (30.99)			135 (27.89)		
**Education level**			3.90	0.272		13.85	0.003
Primary or lower	221 (14.41)	59 (26.70)			47 (21.27)		
Secondary school	340 (22.16)	88 (25.88)			81 (23.82)		
High school/specialized school	309 (20.14)	95 (30.74)			96 (31.07)		
College or higher	664 (43.29)	206 (31.02)			211 (31.78)		
**Occupation**			8.44	0.004		18.35	<0.001
Employed	486 (31.68)	166 (34.16)			173 (35.60)		
Unemployed	1048 (68.32)	282 (26.91)			262 (25.00)		
**BMI** (kg/m^2^)			1.14	0.767		0.91	0.823
Thin (<18.5)	173 (11.28)	50 (28.90)			47 (27.17)		
Normal (18.5–24)	909 (59.25)	262 (28.82)			266 (29.26)		
Overweight (24–28)	358 (23.34)	104 (29.05)			104 (27.09)		
Obese (≥28)	94 (6.13)	32 (34.04)			25 (26.60)		
**Age at initiation of smoking** (years)			0.99	0.321		0.00	0.995
<18	148 (9.65)	38 (25.68)			42 (28.38)		
≥18	1386 (90.35)	410 (29.58)			393 (28.35)		
**Cigarettes smoked on average daily**			20.93	<0.001		23.10	<0.001
≤10	714 (46.54)	247 (34.59)			244 (34.17)		
11–20	676 (44.07)	173 (25.59)			162 (23.96)		
≥21	143 (9.39)	28 (19.44)			29 (20.14)		
**Smoking duration** (years) (median=20.90)			1.13	0.288		1.22	0.270
<21	922 (60.10)	260 (28.20)			271 (29.39)		
≥21	612 (39.90)	188 (30.72)			164 (26.80)		
**Fagerström test for nicotine dependence**			20.66	<0.001		23.89	<0.001
Low (0–3)	591 (38.53)	199 (33.67)			205 (34.69)		
Moderate (4–5)	393 (25.62)	127 (32.32)			111 (28.24)		
Severe (6–10)	550 (35.85)	122 (22.18)			119 (21.64)		
**Previous quit attempts**			3.99	0.046		6.12	0.013
0	937 (61.08)	291 (31.06)			287 (30.63)		
≥1	597 (38.92)	157 (26.30)			148 (24.79)		
**Readiness to quit**			130.61	<0.001		84.72	<0.001
Has started to quit	314 (20.47)	136 (43.31)			124 (39.49)		
Today	432 (28.16)	174 (40.28)			163 (37.73)		
Within 7 days	209 (13.63)	60 (28.71)			55 (26.32)		
Within one month	220 (14.34)	42 (19.09)			42 (20.45)		
After one month	359 (23.40)	36 (10.03)			48 (13.37)		
**Reasons for quitting smoking**			85.55	<0.001		58.21	<0.001
Got illness	469 (30.58)	158 (33.69)			144 (30.70)		
Focus on self and family health	617 (40.22)	203 (32.90)			206 (33.39)		
Affected by the surrounding environment	45 (2.93)	24 (53.33)			17 (37.78)		
Smoke-free ban	12 (0.78)	5 (41.67)			4 (33.33)		
Pregnancy	34 (2.22)	12 (35.29)			10 (29.41)		
Family dissuaded smoking	56 (3.65)	20 (35.71)			21 (37.50)		
Other	301 (19.62)	26 (8.64)			33 (10.96)		
**Intervention**			19.69	<0.001		16.21	<0.001
Counseling	1031 (67.21)	264 (25.61)			259 (25.12)		
Counseling combined with drug therapy	503 (32.79)	184 (36.58)			176 (34.99)		
**Perceived importance of quitting** (median=8.0)			54.97	<0.001		20.63	<0.001
<8.0	680 (44.33)	133 (19.56)			153 (22.50)		
≥8.0	854 (55.67)	315 (36.89)			282 (33.02)		
**Perceived difficulty in quitting** (median=8.0)			6.54	0.011		10.08	0.002
<8.0	737 (48.04)	238 (32.29)			237 (32.16)		
≥8.0	797 (51.96)	210 (26.35)			198 (24.84)		
**Perceived confidence in quitting** (median=6.0)			50.08	<0.001		27.79	<0.001
<6.0	601 (39.18)	114 (18.97)			125 (20.80)		
≥6.0	933 (60.82)	334 (35.80)			310 (33.23)		
**Perceived health status at the first visit**			30.55	<0.001		37.11	<0.001
Very good/good	556 (36.25)	203 (36.51)			195 (35.07)		
Fair	702 (45.76)	194 (27.64)			199 (28.35)		
Very poor/poor	276 (17.99)	51 (18.48)			41 (14.86)		

In addition, there were differences in baseline characteristics between lost female smokers and those who completed the follow-up, mainly due to unable to be contacted. Smokers in the central and western regions, those with a smoking history of <21 years, those who only received counselling, and those who considered their physical condition fair were more likely to be lost to follow-up at one month. The situation in the third month was similar to the first month. Still, the smoking history was no longer related to loss to follow-up, and smokers aged <50 years and those who smoked <20 cigarettes a day, were more likely to be lost to follow-up (Supplementary file Table S1).

### Rates of self-reported 7-day point prevalence abstinence at 1 month

By the end of the first month, 29.20% of outpatients reported they did not smoke within the past seven days (Table 1). The self-reported 7-day PPAR at 1 month varied by region, with the highest rate of 32.06% in the east region, followed by 26.98% in the central region and 22.71% in the west region (p=0.005). Those employed were significantly more likely to quit than the non-employed (34.16% vs 26.91%, p=0.004). We found those who smoked ≤10 cigarettes per day with the highest quitting rate and the lowest for those who smoked ≥21 cigarettes per day (34.59% versus 19.44%, p<0.001). The lower nicotine dependence was associated with higher PPAR at 1 month, with quitting rates of 33.67%, 32.32%, and 22.18% for mild, moderate, and severe nicotine dependence, respectively (p<0.001). Those who never tried to quit had a higher PPAR at 1 month than those who tried in the past (31.06% vs 26.30%, p=0.046). There was a significant correlation between earlier preparation to quit smoking and higher PPAR levels at 1 month in female smokers in SCCs (p<0.001). PPAR was 43.31% among those who had already started quitting and 40.28% among those who were prepared to quit today, while it was only 10.03% among those who intended to quit after one month. Based on quitting reasons, there were significant disparities in quitting rates between the different reasons (p<0.001). Although the proportion of female outpatients quitting because of environmental impacts and smoke-free policies was relatively low, the PPAR at 1 month was comparatively high (53.33% and 41.67%, respectively), followed by family dissuaded smoking (35.71%), pregnancy (35.29%), and considering health (32.90%) and suffering illness (33.69%). Moreover, there was a significant increase in success rate when female smokers received counselling combined with drugs compared to counselling only (36.58% vs 25.61%, p<0.001). For outpatients, the PPAR at 1 month with the score of perceived importance of quitting ≥8.0 was significantly higher than those with an importance score <8.0 points (36.89% vs 19.56%, p<0.001). Compared with those who had quitting difficulty scores ≥8.0, those who scored <8.0 had a higher PPAR at 1 month (32.29% vs 26.35%, p<0.05). The outpatients with a self-reported quit confidence score ≥6.0 were significantly more likely to quit at 1 month than those who scored <6.0 (35.80% vs 18.97%, p<0.001). In addition, according to the perceived health status at the first visit among female outpatients in our SCCs, the PPAR at 1 month among those who believed their health condition to be good (36.51%) was higher than those with fair health (27.64%) and poor health (18.48%) (p<0.001). There was no significant difference in the PPAR at 1 month among age groups, education level, BMI, start age, and duration of smoking (p>0.05).

### Rates of self-reported 7-day point prevalence of abstinence at 3 months

As shown in Table 1, the 7-day point prevalence of abstinence rate was 28.36% at the follow-up at 3 months. Female outpatients from the east had the highest 7-day PPAR at 3 months, followed by those in the west region and then in the central, with 31.31%, 28.08%, and 18.71% (p<0.001), respectively. There was a significant difference in PPAR at follow-up at 3 months by education level, with the highest rate of 31.78% for college or higher, and the lowest rate of 21.27% for primary or lower (p=0.003). The PPAR at 3 months was higher among those who smoked less, and this trend is significant, with the higher quitting rate for those smoking ≤10 cigarettes per day and the lowest for those smoking ≥21 cigarettes per day (34.17% vs 20.14%, p<0.001). At follow-up at 3 months, there were significant differences in quitting reasons according to PPAR, with the higher quitting rate being driven by the surrounding environment and family dissuaded smoking, at 37.78% and 37.50%, respectively, followed by concern about their health and that of the family members (33.39%), smoke-free policies (33.33%), attributed self-illness (30.70%), and pregnancy (29.41%) (p<0.001).

### Rates of self-reported continuous abstinence at 3 months

Among female outpatients, 19.88% reported they had quit continuously up to the follow-up at 3 months (Table 2). There were significant differences in the continuous abstinence rate at 3 months by region, with CAR values of 23.75%, 13.67%, and 13.88% in the east, central, and west region, respectively (p<0.001). CAR at 3 months among those who had better education level was significantly higher than those with lower education level (p=0.002), with those who graduated from high school and college or higher at 23.62% and 22.44%, respectively, and those who graduated from secondary school with the lowest CAR at 14.41%. Compared with female outpatients who were employed, those unemployed had a significantly lower CAR at 3 months (17.27% vs 25.51%, p<0.001). Regarding the number of cigarettes smoked per day, the highest quitting rate was 23.95% for those who smoked ≤10 cigarettes per day, followed by 17.75% and 9.72% for those who smoked 11–20 and ≥21 cigarettes per day (p<0.001). There was a significant difference observed in the dependence on nicotine with a negative dose-response relationship, with CAR being 24.53%, 20.87%, and 14.18% for smokers with low, moderate, and severe nicotine dependence, respectively (p<0.001). Female outpatients who had tried to quit smoking in the past had significantly lower CAR at 3 months compared with those who had never attempted (16.42% vs 22.09%, p=0.006). Similar to the results of the follow-up at 1 month, the earlier the outpatients intended to quit, the significantly higher the CAR (p<0.001). For those who had already started quitting or were ready to quit today, their CAR was 31.21% and 26.85%, respectively, and the lowest CAR (6.69%) was among those who were prepared to quit after 1 month. Among female smokers in SCCs, there was a significant difference in CAR among different quitting reasons (p<0.001), and those who quit as a result of the surrounding environment (33.33%) and smoke-free policies (33.33%) had higher CAR at 3 months, followed by those dissuaded by family (32.14%), concern about their health and that of their family members (23.18%), got illness (21.54%), and pregnancy (20.59%). Looking at the use of intervention methods, the CAR at 3 months of female smokers who received counselling combined with drugs was significantly higher than that of smokers who received counselling only (27.24% versus 16.29%, p<0.001). The CAR at 3 months of female smokers in SCCs with perceived quitting importance scores ≥8.0 was significantly higher than those whose score was <8.0 (24.47% vs 14.12%, p<0.001). Compared with those with quitting difficulty score <8.0, those whose score was ≥8.0 had a lower CAR at 3 months (17.82% vs 22.12%, p=0.035). We also found those female smokers whose confidence in quitting score was ≥6.0 were significantly more likely to quit than others (24.76% vs 12.31%, p<0.001). In addition, the CAR at 3 months was substantially higher among those with good physical condition (26.98%) than those with a fair (18.38%) or poor physical condition (9.42%) (p<0.001). There were no significant differences in the prevalence of continuous abstinence with age, BMI, age at initiation of smoking, and the number of smoking years (p>0.05).

**Table 2:** Self-reported continuous abstinence rate (CAR) of Chinese female smokers at follow-up at 3 months, according to demographic characteristics and tobacco-related factors (N=1534)

	*Smokers n*	*CAR at 3 months n (%)*	*χ^2^*	*p*
**Total**	1534	305 (19.88)		
**Demographic characteristics**				
**Region**			22.72	<0.001
East	939	223 (23.75)		
Central	278	38 (13.67)		
West	317	44 (13.88)		
**Age** (years)			9.41	0.052
18–29	178	43 (24.16)		
30–39	319	53 (16.61)		
40–49	255	41 (16.08)		
50–59	298	71 (23.83)		
≥60	484	97 (20.04)		
**Education level**			14.64	0.002
Primary or lower	221	34 (15.38)		
Secondary school	340	49 (14.41)		
High school/specialized school	309	73 (23.62)		
College or higher	664	149 (22.44)		
**Occupation**			14.16	<0.001
Employed	486	124 (25.51)		
Unemployed	1048	181 (17.27)		
**BMI** (kg/m^2^)			0.06	0.996
Thin (<18.5)	173	35 (20.23)		
Normal (18.5–24)	909	180 (19.80)		
Overweight (24–28)	358	72 (20.11)		
Obese (≥28)	94	18 (19.15)		
**Tobacco-related factors**				
**Age at initiation of smoking** (years)			0.28	0.599
<18	148	27 (18.24)		
≥18	1386	278 (20.06)		
**Cigarettes smoked on average daily**			18.67	<0.001
≤10	714	171 (23.95)		
11–20	676	120 (17.75)		
≥21	143	14 (9.72)		
**Smoking duration** (years) (median=20.90)			0.01	0.929
<21	922	184 (19.96)		
≥21	612	121 (19.77)		
**Fagerström test for nicotine dependence**			19.49	<0.001
Low (0–3)	591	145 (24.53)		
Moderate (4–5)	393	82 (20.87)		
Severe (6–10)	550	78 (14.18)		
**Previous quit attempt**			7.38	0.006
0	937	207 (22.09)		
≥1	597	98 (16.42)		
**Readiness to quit**			84.98	<0.001
Has started to quit	314	98 (31.21)		
Today	432	116 (26.85)		
Within 7 days	209	39 (18.66)		
Within one month	220	28 (12.73)		
After one month	359	24 (6.69)		
**Reasons for quitting smoking**			55.06	<0.001
Self-disease	469	101 (21.54)		
Focus on self and family health	617	143 (23.18)		
Affected by the surrounding environment	45	15 (33.33)		
Smoke-free ban	12	4 (33.33)		
Pregnancy	34	7 (20.59)		
Family dissuaded smoking	56	18 (32.14)		
Other	301	17 (5.65)		
**Intervention**			25.41	<0.001
Counseling	1031	168 (16.29)		
Counseling combined with drug therapy	503	137 (27.24)		
**Perceived importance of quitting** (median=8.0)			25.48	<0.001
<8.0	680	96 (14.12)		
≥8.0	854	209 (24.47)		
**Perceived difficulty in quitting** (median=8.0)			4.44	0.035
<8.0	737	163 (22.12)		
≥8.0	797	142 (17.82)		
**Perceived confidence in quitting** (median=6.0)			35.55	<0.001
<6.0	601	74 (12.31)		
≥6.0	933	231 (24.76)		
**Perceived health status at the first visit**			37.54	<0.001
Very good/good	556	150 (26.98)		
Fair	702	129 (18.38)		
Very poor/poor	276	26 (9.42)		

The 3 months CAR was 33.78%, excluding those who were lost to follow-up (i.e. PP analysis). The results of the PP analyses were similar to those of the primary analyses, except for the differences in residence region and age. The 3 months CAR varied by region, with the smokers from central, eastern and western regions having a 3 months CAR of 36.89%, 35.40% and 25.88%, respectively (p>0.05). Besides, there were significant differences in CAR at 3 months by age (p=0.021), with the highest observed in those aged 18–29 years (36.89%), similar to those aged 50–59 years (36.22%) and 60–69 years (34.04%), and the lowest CAR at 3 months was observed in those aged 40–49 years (28.28%) (Supplementary file Table S2).

The factors selected in the analysis of predictors of quitting include demographic characteristics and tobacco-related factors. Demographic characteristics included residence region, age, education level, occupation, and perceived health status. Tobacco use-related factors included the number of cigarettes smoked per day, FTND, the experience of quitting, readiness to quit, intervention method, perceived importance, difficulty, and confidence in quitting. Since the number of cigarettes smoked per day was a component of FTND, we only used FTND, a more comprehensive indicator, in the stepwise logistic regression model.

As shown in Table 3, region, age, occupation, health status, FTND, time to prepare for quitting, and intervention model were significant independent predictors of CAR at the follow-up at 3 months. Compared to those aged ≥60 years, the AOR was 0.39 (95% CI: 0.23–0.64) for those aged 30–39 years and 0.41 (95% CI: 0.24–0.69) for those 40–49 years. Residence in the central region (AOR=0.47; 95% CI: 0.31–0.73) and west region (AOR=0.48; 95% CI:0.31–0.73), receiving counseling combined with drugs (AOR=2.41; 95% CI:1.73-3.35), non-employment (AOR=0.64; 95% CI: 0.45–0.91), perceived poor health status (AOR=0.37; 95% CI: 0.21–0.64) and fair health status (AOR=0.65; 95% CI= 0.47–0.91), were also independent predictors of quitting. Compared with those with low nicotine dependence, the AOR of successful quitting for female smokers with moderate and severe nicotine dependence was 0.64 (95% CI: 0.44–0.92) and 0.50 (95% CI: 0.34–0.72). In addition, the predictors of quitting by per-protocol (PP) were similar to the results from ITT, except for region and reasons for quitting smoking. By PP, compared to the east region, the AOR of those from the central region and west region was 0.95 (95% CI: 0.56–1.61) and 0.47 (95% CI: 0.29–0.77). Quitting smoking as a result of the surrounding environment (AOR=4.14; 95% CI: 1.27–13.44) was also a strong independent predictor of quitting (Supplementary file Table S3).

**Table 3:** Multiple logistic regression analysis for predictors of continuous abstinence at 3 months among Chinese female smokers

*Predictors*	*AOR (95%CI)*	*p*
**Region**		
East (Ref.)	1	
Central	0.47 (0.31–0.73)	<0.001
West	0.48 (0.31–0.73)	<0.001
**Age** (years)		
≥60 (Ref.)	1	
50–59	0.90 (0.58–1.39)	0.619
40–49	0.41 (0.24–0.69)	<0.001
30–39	0.39 (0.23–0.64)	<0.001
18–29	0.67 (0.38–1.16)	0.148
**Occupation**		
Employed (Ref.)	1	
Unemployed	0.64 (0.45–0.91)	0.012
**Perceived health status at the first visit**		
Very good/good (Ref.)	1	
Fair	0.65 (0.47–0.91)	0.011
Very poor/poor	0.37 (0.21–0.64)	<0.001
**Fagerström test for nicotine dependence**		
Low (0–3) (Ref.)	1	
Moderate (4–5)	0.64 (0.44–0.92)	0.016
Severe (6–10)	0.50 (0.34–0.72)	<0.001
**Readiness to quit**		
After 1 month (Ref.)	1	
Within 1 month	1.79 (0.94–3.43)	0.079
Within 7 days	2.86 (1.53–5.32)	0.001
Today	4.01 (2.35–6.85)	<0.001
Has started to quit	7.11 (4.12–12.27)	<0.001
**Intervention**		
Counseling (Ref.)	1	
Counseling combined with drug therapy	2.41 (1.73–3.35)	<0.001

AOR: adjusted odds ratio; adjusted for region, age, education level, occupation, health status, Fagerström test for nicotine dependence (FTND), the experience of quitting, readiness to quit, intervention method, perceived importance, difficulty, and confidence in quitting.

## DISCUSSION

To our knowledge, this study is the first full-scale evaluation of the efficacy of smoking cessation clinics in China for female smokers and where predictors of successful smoking cessation among female smokers were identified. According to our study, the PPAR at follow-up at 1 month was 29.20% by intention to treat analysis, which was significantly higher than in Hong Kong communities (8.5%) where female smokers were provided with brief smoking cessation intervention through trained ladies^25^, and lower than the 273 SCCs in the mainland of China (34.1%) found by Xie et al.^26^; this may be contributed by the gender difference, as 96.1% of the outpatients studied were male. It indicates that women might be less likely to succeed at quitting than men. In addition, CAR at follow-up at 3 months was 19.88%, similar to that found in a rehabilitation center where smokers received seven counseling intensive interventions (19.7%)^27^, and lower than that reported by female smokers who used UK smoking cessation services (25.5%)^28^. In the study conducted in UK, all subjects were provided with medication, and that might be the reason why it had a higher cessation rate. It also indicates that providing more female smokers with medication could boost smoking cessation rate in SCCs.

In our study, we found the quitting rate was higher in the east regions than in central and western regions at follow-up at 1 month and at 3 months. For example, the CARs at 3 months in the east, central, and west regions were 23.75%, 13.67%, and 13.88%, respectively. Compared with the east regions, the AOR of the central regions was 0.47 (95% CI: 0.31–0.73), and the AOR of the west region was 0.48 (95% CI: 0.31–0.73) from the results of the multivariate analysis. That might be because SCCs in the east region had a higher follow-up rate than in central and west regions, while follow-up time is an independent predictor of smoking cessation success^19^.

Previous studies have proved that the earlier someone quits smoking, the more benefits it brings^29-31^. The study by Jha et al.^11^ shows that quitting smoking before the age of 40 years can reduce 90% of the risk of suffering tobacco-related illness. However, only 32.40% of those who sought professional help from SCCs were aged <40 years, so more young female smokers should be encouraged to attend the SCCs. We also observed that the CARs at 3 months among those aged 30–39 years (AOR=0.39; 95% CI: 0.23–0.64) and 40–49 years (AOR=0.41; 95% CI: 0.24–0.69) were lower than those aged ≥60 years, probably because most women at that stage were focusing on their careers and were exposed to more stress and anxiety, which led to more difficulties in quitting^32,33^. Thus, we should provide intensive psychological interventions for women smokers aged 30–49 years, increase the number and duration of counselling sessions, and encourage the use of smoking cessation drugs.

Female outpatients with moderate and severe nicotine dependence had lower PPAR and CAR at the follow-up visits than those with low nicotine dependence. The smokers’ PPAR at follow-up at 1 month with low, moderate, and severe nicotine dependence was 33.67%, 32.32%, and 22.18%, respectively. For CAR at 3 months, those with moderate (AOR=0.64; 95% CI: 0.44–0.92) and severe (AOR=0.50; 95% CI: 0.34–0.72) nicotine dependence are more likely to fail than those who have a low nicotine dependence, in alignment with other studies^30,34^. This may be attributed to smokers with moderate to severe nicotine dependence being more likely to suffer from severe withdrawal symptoms and stronger tobacco cravings, hence the higher likelihood of relapse.

Similar to the results from the 2018 GATS China survey^7^, our study found that highly educated female smokers were more likely to visit cessation clinics. However, female outpatients with high educational level did not show a higher quitting rate when using the same interventions than those with lower education level. Remarkably, female smokers were less likely to quit than men out of concerns about their health, in agreement with other findings^35^. Compared with those employed, unemployed female smokers had a poor quitting outcome (AOR=0.64, 95% CI: 0.45–0.91), similar to a previous study^26^.

Furthermore, similar to other studies^29,31^, our research found that female smokers who prepared to quit earlier were more likely to quit. The PPAR at follow-up at 1 month was relatively higher in outpatients who quit smoking or choose to quit today (43.31% and 40.28%) than those who decided to quit within 7 days (28.71%), within 1 month (19.09%) and after 1 month (10.03%). For CAR at 3 months, compared with those who were prepared to quit after 1 month, outpatients prepared to quit within 1 month (AOR=1.79; 95% CI: 0.94–3.43), within 7 days (AOR=2.86; 95% CI: 1.53–5.32), today (AOR=4.01; 95% CI: 2.35–6.85), and have started to quit (AOR=7.11; 95% CI: 4.12–12.27), were more likely to quit smoking successfully.

Many studies indicated that the quitting rate could be significantly improved through drug therapy^26^, and so did our analysis. The PPARs at follow-up at 1 month of outpatients who received counselling combined with drugs and those who only received counselling were 36.58% and 25.61%, respectively. From the logistic regression model results, counselling combined with drug therapy (AOR=2.41; 95% CI: 1.73–3.35) can significantly improve the quitting rate than counselling only. Unfortunately, while physicians in SCCs will recommend drugs to all smokers for quitting, only 32.79% of outpatients used drugs in our study. This could be attributed to the difficulties some female smokers experienced with the availability and affordability of drugs in the hospitals. Thus, we strongly suggest that more SCCs should provide cessation medications and compensation to reduce the burden of taking smoking cessation medications and facilitate medication uptake.

In the present study, outpatients with good, fair, and poor perceived physical condition, the PPARs at follow-up at 1 month were 36.51%, 27.64%, and 18.48%, respectively, and we also found that the CAR at 3 months of outpatients with fair (AOR=0.65; 95% CI: 0.47–0.90) and poor (AOR=0.37; 95% CI: 0.21–0.64) perceived physical condition were more likely to fail at quitting than those in good physical condition. This may be because, in our study, 74.64% of female smokers with poor health status were found to be heavily addicted to nicotine, and those who are heavily addicted to nicotine have more difficulty in quitting smoking^30^. Additionally, the low proportion of cessation clinics providing drugs might be another potential reason. Therefore, there is a need for a focus on female smokers with poor health status in smoking cessation clinics, such as providing more frequent counselling services and medications to them.

### Limitations

The present study has some limitations. First, the quit rate was based on self-report, lacking biochemical validation. Second, there are no medium- and long-term smoking cessation assessments such as follow-up at 6 months and 12 months because China’s management of SCC requires only follow-up at 1 month and at 3 months. Finally, we regard the lost female smokers as failing to quit, which may underestimate the quit rate in the SCCs.

## CONCLUSIONS

Our research found a significant regional disparity in the quit rates of female smokers using smoking cessation clinics, with higher quit rates among smokers in the east region. It indicates that capacity building for physicians of SCCs in the west and central regions should be strengthened. Smokers with high nicotine dependence, those unemployed, and those in poor health condition are less likely to be successful in quitting. In contrast, those who intended to quit earlier, those who use counselling combined with drug therapy, and those aged >60 years are more likely to quit successfully. Highly educated female smokers were more likely to visit cessation clinics but high education level did not show a significant association with a higher quitting rate. Our study suggests that efforts should be taken to improve female smokers’ motivation to quit and intensive psychological interventions should be provided, especially for young women. Besides, if more SCCs could be equipped with cessation medication, it might assist female smokers to quit.

## Data Availability

The data supporting this research are available from the authors on reasonable request.
