# Supplementary material for: Predictors for quitting smoking in smoking cessation clinics among female smokers in China
Source: Tob Induc Dis. 2023 Feb 17;21:26. doi: 10.18332/tid/159132 (PMC9936604; doi:10.18332/tid/159132)
Supplement: Supplementary file 1 [file TID-21-26-s1.pdf]

Table S1. Demographic characteristics and tobacco related factors of completed and lost to 1-month follow-up and 3-month follow-up among Chinese female smokers (N=1534)

	N (%)	Completed *n* (%)	Uncompleted *n (%)*	X^2	*P-value*	Completed *n* (%)	Uncompleted n (%)	X^2	*P-value*
	1534	1154(75.23)	380(24.77)			903(58.87)	631(41.13)		
Demographic characteristics									
Region				75.86	<0.001			84.48	<0.001
East	939(61.21)	777(82.75)	162(17.25)			630(67.09)	309(32.91)		
Central	278(18.12)	168(60.43)	110(39.57)			103(37.05)	175(62.95)		
West	317(20.67)	209(65.93)	108(34.07)			170(53.63)	147(46.37)		
Age (years)				8.96	0.062			10.18	0.038
18-29	178(11.60)	129(72.47)	49(27.53)			92(51.69)	86(48.31)		
30-39	319(20.80)	228(71.47)	91(28.53)			185(57.99)	134(42.01)		
40-49	255(16.62)	184(72.16)	71(27.84)			145(56.86)	110(43.14)		
50-59	298(19.43)	237(79.53)	61(20.47)			196(65.77)	102(34.23)		
60+	484(31.55)	376(77.69)	108(22.31)			285(58.88)	199(41.12)		
Education				6.92	0.075			2.55	0.466
Primary and below	221(14.41)	175(79.19)	46(20.81)			132(59.73)	89(40.27)		
Secondary school	340(22.16)	258(75.88)	82(24.12)			193(56.76)	147(43.24)		
High school/specialized school	309(20.14)	242(78.32)	67(21.68)			193(62.46)	116(37.54)		

	N (%)	Completed *n* (%)	Uncompleted *n (%)*	X^2	*P-value*	Completed *n* (%)	Uncompleted n (%)	X^2	*P-value*
College and above	664(43.29)	479(72.14)	185(27.86)			385(57.98)	279(42.02)		
Occupation				0.09	0.761			0.779	0.378
Employed	486(31.68)	368(75.72)	118(24.28)			294(60.49)	192(39.51)		
Unemployed	1048(68.32)	786(75.00)	262(25.00)			609(58.11)	439(41.89)		
Body mass index (kg/m2)				5.99	0.112			5.44	0.142
Underweight (<18.5)	173(11.28)	122(70.52)	51(29.48)			89(51.45)	84(48.55)		
Normal (18.5-24)	909(59.25)	691(76.02)	218(23.98)			548(60.29)	361(39.71)		
Overweight (24-28)	358(23.34)	263(73.46)	95(26.54)			207(57.82)	151(42.18)		
Fat (≥28)	94(6.13)	78(82.98)	16(17.02)			59(62.77)	35(37.23)		
Age at initiation of smoking (years)				0.219	0.640			0.46	0.496
<18	148(9.65)	109(73.65)	39(26.35)			91(61.49)	57(38.51)		
≥18	1386(90.35)	1045(75.40)	341(24.60)			812(58.59)	574(41.41)		
Cigarettes smoked on average daily (cig/d)				2.44	0.295			10.08	0.007
≤10	714(46.54)	532(74.51)	182(25.49)			419(58.68)	295(41.32)		
11-20	676(44.07)	506(74.85)	170(25.15)			382(56.51)	294(43.49)		
≥21	143(9.39)	116(80.56)	28(19.44)			102(70.83)	42(29.17)		
Smoking duration (years)				13.66	<0.001			2.44	0.118
<21	922(60.10)	663(71.91)	259(28.09)			528(57.27)	394(42.73)		

	N (%)	Completed *n* (%)	Uncompleted *n (%)*	X^2	*P-value*	Completed *n* (%)	Uncompleted n (%)	X^2	*P-value*
≥21	612(39.90)	491(80.23)	121(19.77)			375(61.27)	237(38.73)		
Fagerström Test for Nicotine Dependency				5.21	0.074			2.95	0.229
Low (0-3)	591(38.53)	451(76.31)	140(23.69)			352(59.56)	239(40.44)		
Moderate (4-5)	393(25.62)	307(78.12)	86(21.88)			242(61.58)	151(38.42)		
Severe (6-10)	550(35.85)	396(72.00)	154(28.00)			309(56.18)	241(43.82)		
Previous quit attempt				0.12	0.726			0.028	0.867
0	937(61.08)	702(74.92)	235(25.08)			550(58.70)	387(41.30)		
≥1	597(38.92)	452(75.71)	145(24.29)			353(59.13)	244(40.87)		
Readiness to quit				6.15	0.188			7.23	0.124
Has started to quit	314(20.47)	241(76.75)	73(23.25)			188(59.87)	126(40.13)		
Today	432(28.16)	334(77.31)	98(22.69)			272(62.96)	160(37.04)		
Within 7 days	209(13.63)	148(70.81)	61(29.19_			112(53.59)	97(46.41)		
Within one month	220(14.34)	156(70.91)	64(29.09)			120(54.55)	100(45.45)		
After one month	359(23.40)	275(76.60)	84(23.40)			211(58.77)	148(41.23)		
Reasons for quitting smoking				9.42	0.151			4.80	0.570
Got illness	469(30.58)	361(76.97)	108(23.03)			286(60.98)	183(39.02)		
Focus on self and family health	617(40.22)	455(73.74)	162(26.26)			357(57.86)	260(42.14)		
Affected by the surrounding environment	45(2.93)	35(77.78)	10(22.22)			21(46.67)	24(53.33)		

	N (%)	Completed *n* (%)	Uncompleted *n (%)*	X^2	*P-value*	Completed *n* (%)	Uncompleted n (%)	X^2	*P-value*
Smoke-free ban	12(0.78)	11(91.67)	1(8.33)			8(66.67)	4(33.33)		
Pregnancy	34(2.22)	24(70.59)	10(29.41)			19(55.88)	15(44.12)		
Family dissuaded smoking	56(3.65)	35(62.50)	21(37.50)			31(55.36)	25(44.64)		
Others	301(19.62)	233(77.41)	68(22.59)			181(60.13)	120(39.87)		
Intervention				3.86	0.049			11.67	<0.001
Counseling	1031(67.21)	760(73.71)	271(26.29)			576(55.87)	455(44.13)		
Counseling combined with drug therapy	503(32.79)	394(78.33)	109(21.67)			327(65.01)	176(34.99)		
Perceived importance of quitting (M=8.0)				2.23	0.135			0.20	0.654
<8.0	680(44.33)	499(73.38)	181(26.62)			396(58.24)	284(41.76)		
≥8.0	854(55.67)	655(76.70)	199(23.30)			507(59.37)	347(40.63)		
Perceived difficulty in quitting (M=8.0)				3.78	0.052			0.19	0.666
<8.0	737(48.04)	538(73.00)	616(77.29)			438(59.43)	299(40.57)		
≥8.0	797(51.96)	199(27.00)	181(22.71)			465(58.34)	332(41.66)		
Perceived confidence in quitting (M=6.0)				2.07	0.150			1.97	0.160
<6.0	601(39.18)	464(77.20)	137(22.80)			367(61.06)	234(38.94)		
≥6.0	933(60.82)	690(73.95)	243(26.05)			536(57.45)	397(42.55)		
Perceived health status at the first visit				18.52	<0.001			17.76	<0.001
Very good/good	556(36.25)	428(76.98)	128(23.02)			337(60.61)	219(39.39)		

	N (%)	Completed<br>*n* (%)	Uncompleted<br>*n (%)*	X^2	*P-value*	Completed<br>*n* (%)	Uncompleted<br>n (%)	X^2	*P-value*
General	702(45.76)	496(70.66)	206(29.34)			378(53.85)	324(46.15)		
Very poor/poor	276(17.99)	230(83.33)	46(16.67)			188(68.12)	88(31.88)		

Table S2. Demographic characteristics and related factors of tobacco use among female smokers during the 3-month follow-up among Chinese female smokers (N=903) by per-protocol

	N (%)	3-month CAR*<br>*n* (%)	X^2	*P-value*
	903	305(33.78)		
Demographic characteristics				
Region			5.92	0.052
East	630(69.77)	223(35.40)		
Central	103(11.41)	38(36.89)		
West	170(18.82)	44(25.88)		
Age (years)			11.58	0.021
18-29	92(10.19)	43(46.74)		
30-39	185(20.49)	53(28.65)		
40-49	145(16.06)	41(28.28)		

		3-month CAR*		
	[illegible]		[illegible]	[illegible]
50-59	196(21.70)	71(36.22)		
60+	285(31.56)	97(34.04)		
Education			15.45	0.002
Primary and below	132(14.62)	34(25.76)		
Secondary school	193(21.37)	49(25.39)		
High school/specialized school	193(21.37)	73(37.82)		
College and above	385(42.64)	149(38.70)		
Occupation			13.75	<0.001
Employed	294(32.56)	124(42.18)		
Unemployed	609(67.44)	181(29.72)		
Body mass index (kg/m2)			1.81	0.612
Underweight (<18.5)	89(9.86)	35(39.33)		
Normal (18.5-24)	548(60.69)	180(32.85)		
Overweight (24-28)	207(22.92)	72(34.78)		
Fat (≥28)	59(6.53)	18(30.51)		
Age at initiation of smoking (years)			0.76	0.383
<18	91(10.08)	27(29.67)		
≥18	812(89.92)	278(34.24)		
Cigarettes smoked on average daily (cig/d)			28.56	<0.001

	3-month CAR*			
≤10	419(46.40)	171(40.81)		
11-20	382(42.30)	120(31.41)		
≥21	102(11.30)	14(13.73)		
Smoking duration (years, M=20.90)			0.65	0.419
<21	528(58.47)	184(34.85)		
≥21	375(41.53)	121(32.27)		
Fagerström Test for Nicotine Dependency			18.72	<0.001
Low (0-3)	352(38.98)	145(41.19)		
Moderate (4-5)	242(26.80)	82(33.88)		
Severe (6-10)	309(34.22)	78(25.24)		
Previous quit attempt			9.37	0.002
0	550(60.91)	207(37.64)		
≥1	353(39.09)	98(27.76)		
Readiness to quit			91.12	<0.001
Has started to quit	188(20.82)	98(52.13)		
Today	272(30.12)	116(42.85)		
Within 7 days	112(12.40)	39(34.82)		
Within one month	120(13.29)	28(23.33)		

		3-month CAR*		
After one month	211(23.37)	24(11.37)		
Reasons for quitting smoking			77.22	<0.001
Self-disease	286(31.67)	101(35.31)		
Focus on self and family health	357(39.54)	143(40.06)		
Affected by the surrounding environment	21(2.33)	15(71.43)		
Smoke-free ban	8(0.89)	4(50.00)		
Pregnancy	19(2.10)	7(36.84)		
Family dissuaded smoking	31(3.43)	18(58.06)		
Others	181(20.04)	17(9.39)		
Intervention			15.11	<0.001
Counseling	576(63.79)	168(29.17)		
Counseling combined with drug therapy	327(36.21)	137(41.90)		
Perceived importance of quitting (M=8.0)			28.66	<0.001
<8.0	396(43.85)	96(24.24)		
≥8.0	507(56.15)	209(41.22)		
Perceived difficulty in quitting (M=8.0)			4.50	0.034
<8.0	438(48.50)	163(37.21)		

	3-month CAR*			
	[illegible]	[illegible]	[illegible]	[illegible]
≥8.0	465(51.50)	142(30.54)		
Perceived confidence in quitting (M=6.0)			51.22	<0.001
<6.0	367(40.64)	74(20.16)		
≥6.0	536(59.36)	231(43.10)		
Perceived health status at the first visit			50.82	<0.001
Very good/good	337(37.32)	150(44.51)		
General	378(41.86)	129(34.13)		
Very poor/poor	188(20.82)	26(13.83)		

*CAR: Continuous abstinence rate.

Table S3. Logistic regression analysis for predictors of quitting at 3-month follow-up among Chinese female smokers (N=903) by per-protocol

**Predictors**	***AOR*** **(95%*****CI*****)**	***P-value***
3-month CAR		
Region		
East (Ref.)	1	
Central	0.95(0.56-1.61)	0.840
West	0.47(0.29-0.77)	0.002
Age (years)		
60+ (Ref.)	1	
50-59	0.77(0.46-1.29)	0.328
40-49	0.39(0.21-0.73)	0.003
30-39	0.32(0.18-0.59)	<0.001
18-29	0.74(0.37-1.47)	0.387
Occupation		
Employed (Ref.)	1	
Unemployed	0.65(0.43-0.99)	0.045
Perceived health status at the first visit		
Very good/good (Ref.)	1	
General	0.65(0.43-0.97)	0.036
Very poor/poor	0.34(0.18-0.64)	<0.001
FTND		
Low (0-3) (Ref.)	1	

Predictors	*AOR* (95%*CI*)	*P-value*
Moderate (4-5)	0.59(0.38-0.91)	0.016
Severe (6-10)	0.42(0.28-0.65)	<0.001
Readiness to quit		
After 1 month (Ref.)	1	
Within 1 month	1.15(0.42-3.13)	0.792
Within 7 days	2.19(0.80-5.97)	0.127
Today	2.58(1.02-6.51)	0.046
Has started to quit	4.58(1.77-11.87)	0.002
Reasons for quitting smoking		
Self-disease (Ref.)	1	
Focus on self and family health	0.86(0.55-1.37)	0.529
Affected by the surrounding environment	4.14(1.27-13.44)	0.018
Smoke-free ban	1.17(0.18-2.15)	0.875
Pregnancy	0.63(0.18-2.15)	0.459
Family dissuaded smoking	2.00(0.80-5.00)	0.139
Others	0.55(0.19-1.59)	0.269
Intervention		
Counseling (Ref.)	1	
Counseling combined with drug therapy	2.96(1.97-4.44)	<0.001
Perceived confidence in quitting (M=6.0)		
<6.0 (Ref.)	1	

Predictors	*AOR* (95%*CI*)	*P-value*
≥6.0	1.75(1.17-2.64)	0.007

*AOR: adjusted odds ratio; adjusted for region, age, education level, occupation, health status, Fagerström Test for Nicotine Dependence (FTND), the experience of quitting, readiness to quit, intervention method, perceived importance, difficulty, and confidence in quitting

© 2023 Lin B. et al.
